# ESR Essentials: imaging of middle ear cholesteatoma—practice recommendations by the European Society of Head and Neck Radiology

**DOI:** 10.1007/s00330-024-11021-x

**Published:** 2024-08-26

**Authors:** Philip Touska, Steve E. J. Connor

**Affiliations:** 1 Department of Radiology, Guy’s and St. Thomas’ NHS Foundation Trust, Guy’s Hospital, Great Maze Pond, London, SE1 9RT United Kingdom; 2 Department of Neuroradiology, Kings College Hospital NHS Trust, Denmark Hill, London, SE5 9RS United Kingdom; 3 School of Biomedical Engineering & Imaging Sciences Clinical Academic Group, King’s College London, London, United Kingdom

**Keywords:** Cholesteatoma ear, Middle, Diffusion magnetic resonance imaging, Tomography (X-ray computed), Ear diseases

## Abstract

**Abstract:**

Although non-malignant, middle ear cholesteatoma can result in significant complications due to local bone erosion and infection. The treatment of cholesteatoma is surgical, but residual disease is common and may be clinically occult, particularly when the canal wall is preserved or reconstructive techniques are employed. Imaging plays a pivotal role in the management of patients with middle ear cholesteatoma—aiding clinical diagnosis, identifying complications, planning surgery, and detecting residual disease at follow-up. Computed tomography is the primary imaging tool in the preoperative setting since it can provide both a surgical roadmap and detect erosive complications of cholesteatoma. The ability of magnetic resonance imaging with non-echoplanar diffusion-weighted sequences to accurately detect residual disease has led to a shift in the diagnostic paradigm for post-surgical follow-up of cholesteatoma, such that routine “second-look” surgery is no longer required. The following practice recommendations are aimed at helping the radiologist choose appropriate imaging approaches and understand the key diagnostic considerations for the evaluation of pre- and post-surgical middle ear cholesteatoma.

**Key Points:**

- *In the preoperative setting, CT is the first-line imaging modality and MRI is reserved for rare clinical scenarios (low evidence)*.
- *Non-echoplanar imaging (EPI) DWI is the optimal MRI sequence for the detection of residual cholesteatoma (moderate evidence)*.
- *Non-EPI DWI plays an important role in the postoperative surveillance of cholesteatoma (moderate evidence)*.

## Key recommendations

- Diagnosis and preoperative planning: high-resolution CT can be used to clarify clinical diagnosis, detect complications, and delineate surgical anatomy; MRI is only indicated in very specific situations (low evidence).
- MRI technique: non-echoplanar imaging (EPI) (or multi-shot EPI) DWI sequences should be preferentially acquired over single-shot EPI DWI sequences, and they are the optimal imaging technique for cholesteatoma detection (moderate evidence).
- Residual disease: non-EPI (or multi-shot EPI) DWI with supplementary anatomical sequences plays a key role in imaging surveillance of postoperative cholesteatoma and this should be continued for at least 5 years (moderate evidence).

## Introduction

Cholesteatomas comprise desquamated keratin and squamous debris surrounded by a fibrous matrix. They most commonly occur within the middle ear and affect approximately 6–9 per 100,000 individuals in developed countries [1].

Although non-neoplastic, the matrix and perimatrix surrounding cholesteatomas can stimulate a variably aggressive inflammatory response that may result in extensive bone erosion with injury to critical temporal bone structures (e.g., the facial nerve and inner ear) and intracranial extension.

Cholesteatomas are usually diagnosed clinically and treated surgically. Imaging plays a key role in the evaluation of middle ear cholesteatoma, supporting the clinical diagnosis, demonstrating complications, planning surgery, and detecting residual disease at follow-up.

This article provides practice recommendations for the appropriate imaging approaches and key diagnostic considerations in the evaluation of pre- and post-surgical middle ear cholesteatoma.

## Background

### Classification

**Fig. 1:**
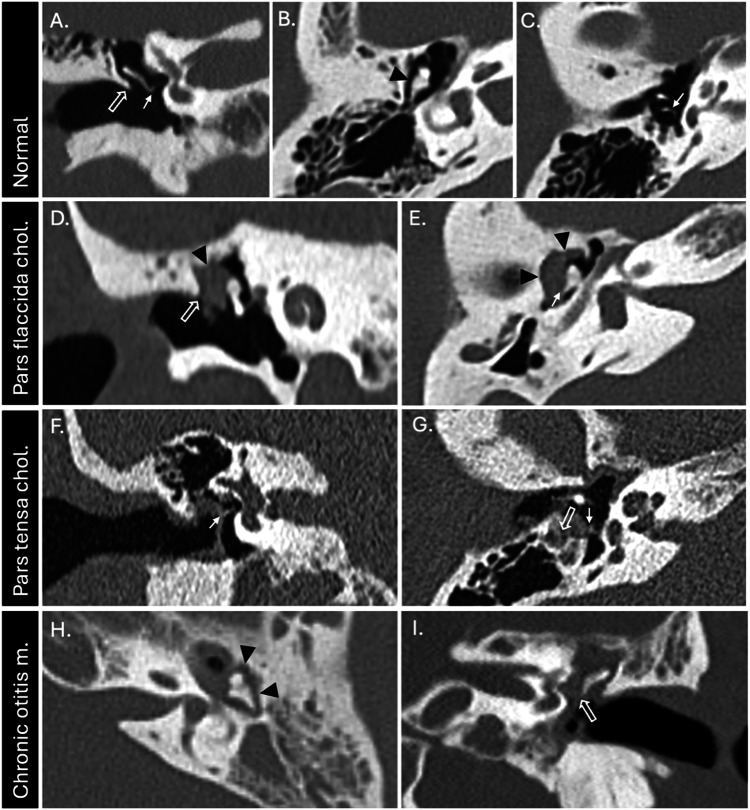
Appearances of the ossicles and scutum in a healthy temporal bone, pars flaccida cholesteatoma, pars tensa cholesteatoma, and chronic otitis media (without cholesteatoma). Coronal (**A**) and axial (**B**, **C**) sections through a healthy right temporal bone showing an intact scutum (outlined arrow), incudostapedial articulation (small arrow) and aerated lateral epitympanum (black arrowhead). Coronal (**D**) and axial (**E**) sections through the right temporal bone of a patient with a pars flaccida cholesteatoma showing non-dependent soft tissue filling the lateral epitympanum (black arrowheads), causing scutal erosion (outlined arrow) and incudal erosion (small arrow). Coronal (**F**) and axial (**G**) sections through the right temporal bone of a patient with a pars tensa cholesteatoma showing erosion of the long process of the incus, but preservation of the stapes (small arrow) and extension to the facial recess (outlined arrow). Axial (**H**) and coronal (**I**) sections through the left temporal bone of a patient with chronic otitis media without cholesteatoma showing opacification of the lateral epitympanum without local bone erosion (black arrowheads), but there is attenuation of the distal long process of the incus at the level of a pars tensa perforation (outlined arrow)

Cholesteatomas can be classified according to their aetiology as congenital or acquired. Congenital cholesteatomas are uncommon (4–24% of cholesteatomas) and are thought to arise from persistent rests or entrapped foci of squamous epithelium within the middle ear cavity behind an intact tympanic membrane [1, 2]. Acquired cholesteatomas are more common and arise later in life. They most frequently develop in the context of chronic suppurative otitis media (CSOM) which is characterised by chronic ear discharge (otorrhea). They can be further subclassified into those related to a retraction pocket (primary acquired cholesteatoma) or those related to a perforation (secondary acquired cholesteatoma). Alternatively, acquired cholesteatomas may be classified according to their sites of origin as pars flaccida (approximately 80% of cases) or pars tensa (approximately 20% of cases) cholesteatomas with specific erosion and extension patterns (Fig. 1) [3].

### Surgical management

**Fig. 2:**
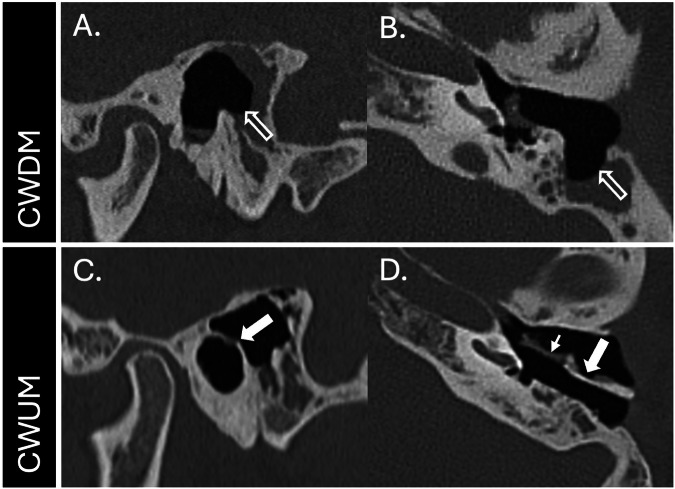
Mastoidectomy types. Sagittal (**A**) and axial (**B**) reconstructions from an unenhanced CT through the left temporal bone of a patient who has previously undergone CWDM. There is deficiency of the posterior EAC wall and the formation of a cavity (outlined arrows), which needs to be cleaned under otoscopic guidance. Sagittal (**C**) and axial (**D**) CT reconstructions in another patient who has previously undergone a CWUM with an intact posterior EAC wall (thick arrows). A defect in the superomedial EAC wall and tympanic membrane has been repaired with tragal cartilage (small arrow)

The most common means of removing a cholesteatoma is to use a retro-auricular approach. This can be defined according to whether the posterior external auditory canal (EAC) wall is removed (canal wall down mastoidectomy or CWDM) or preserved (canal wall up mastoidectomy or CWUM) (Fig. 2). A CWDM is effective at eradicating cholesteatoma, but results in a large cavity that needs frequent cleaning, maintenance of water precautions, and results in difficulties fitting hearing aids [4]. A CWUM avoids these disadvantages, but due to greater preservation of bony structures, complete removal of cholesteatoma is more challenging and CWUMs are associated with higher residual disease rates [5]. In order to mitigate the disadvantages of CWDM, the posterior EAC wall can be reconstructed and mastoid obliterated using a variety of materials, including cartilage, bone (powder, chips, or pâté), fascia, or hydroxyapatite [5].

For smaller cholesteatomas, minimally invasive endaural or transcanal approaches can be employed with limited bone resection (atticotomy or atticoantrostomy). Increasingly, such techniques are being performed endoscopically and accurate delineation of lesion extent is critical to ensure appropriate patient selection and to avoid conversion to open surgery [6].

Where cholesteatoma is extensive and involves the petrous apex, more radical surgery, such as a subtotal petrosectomy (where all mastoid cells are exteriorised and middle ear epithelium, facial nerve canal and otic capsule are removed) may be required. Such surgical defects are packed with material such as abdominal fat and the EAC is closed (blind-sac closure).

Following removal of cholesteatoma, the ossicular chain may require reconstruction (ossiculoplasty) using autologous grafts or ossicular replacement prostheses, and any defects in the tympanic membrane can be reconstructed with fascia or cartilage [5].

### Post-surgical residual or recurrent cholesteatoma

Recurrent cholesteatoma results from the reformation of a retraction pocket, whereas residual cholesteatoma results from incomplete removal of the cholesteatoma matrix. In adults, the surgical approach significantly influences the risk of residual disease with higher rates following CWUM (9–70%) compared with CWDM (5–17%) [7]. In children, rates of residual disease are relatively high (16–54%), but the surgical approach does not appear to exert a significant influence [8]. Nevertheless, where the EAC wall is preserved, any residual cholesteatoma is typically clinically occult. As a result, prior to the widespread use of MRI surveillance, patients undergoing CWUM would require a second-look operation 9–12 months after the initial surgery to identify and remove any residual disease. Rates of residual disease can be reduced through the use of endoscopic approaches, which provide views of surgical ‘blind spots’ such as the anterior epitympanic (supratubal) recess, facial recess, or sinus tympani, facilitating removal [9]. Mastoid obliteration techniques can also reduce the risk of recidivism, but as with other techniques where anatomy (including the drumhead) is either preserved or reconstructed, MRI surveillance is needed to detect occult disease [10].

## Imaging modalities

### CT

#### Multidetector CT (MDCT)

**Fig. 3:**
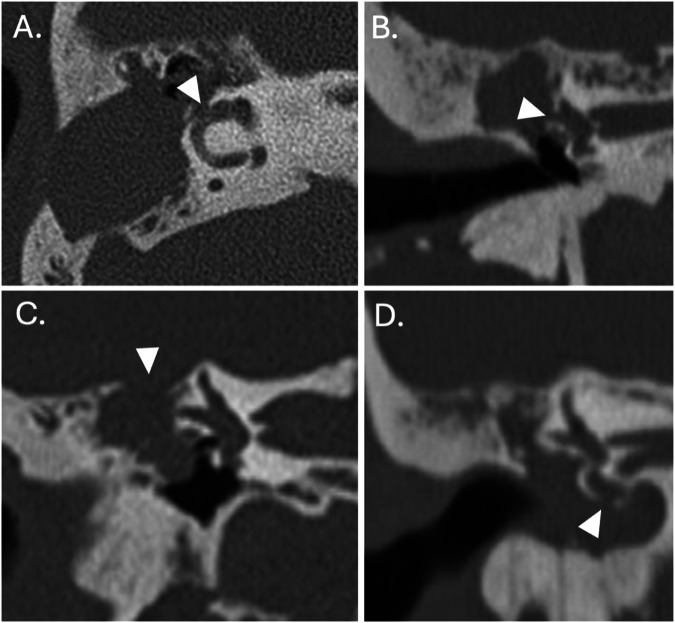
Cholesteatoma-mediated bone erosion on CT. Axial (**A**) and coronal (**B**) CT sections through the right temporal bone of a patient with an extensive tympanomastoid cholesteatoma demonstrating erosion of the lateral semicircular canal (arrowheads). Coronal CT reconstruction (**C**) in another patient with an extensive right epitympanic cholesteatoma demonstrating tegmental erosion (arrowhead). A coronal reconstruction (**D**) in a different patient with an extensive middle ear cholesteatoma demonstrates erosion of the basal turn of the cochlea (arrowhead)

Contemporary CT scanners, with beam collimation widths of 0.5–0.75 mm, can depict temporal bone anatomy and cholesteatoma-induced erosion (Fig. 3). Helically acquired datasets should be reconstructed using overlapping sections with the aid of appropriate bone algorithms. The relatively higher radiation doses needed to provide high-resolution temporal bone imaging can be partially mitigated through the use of iterative reconstruction techniques [11].

#### Cone beam CT (CBCT), ultra-high-resolution CT (U-HRCT), and photon-counting detector CT (PCD-CT)

CBCT can provide higher resolution (0.15–0.5 mm) isotropic imaging with lower radiation doses than MDCT [12]. U-HRCT also provides high spatial resolution (e.g., slice thicknesses of 0.25 mm), but requires the application of advanced noise reduction algorithms to mitigate radiation dose increases [13]. Both techniques can offer superior delineation of temporal bone anatomy; however, for CBCT, longer acquisition times make it prone to motion artefacts that can limit its use in some patients (e.g., younger children). Nevertheless, the limitations of CBCT can be mitigated by a combination of good immobilisation, supine positioning and more powerful x-ray sources [12]. The use of dedicated CBCT systems with wide fields of view is also critical since not all CBCT systems on the market are capable of dedicated temporal bone imaging. PCD-CT provides very high spatial resolution with lower image noise and has been shown to provide superior delineation of temporal bone anatomy when compared to MDCT [14]. Whilst PCD-CT is not yet in widespread use, it may be helpful in assessing the ossicular chain and detecting early bone erosion in cholesteatoma.

#### Optimal CT modality

There is currently insufficient data to establish the superiority of CBCT, U-HRCT and PCD-CT over MDCT in the setting of routine clinical cholesteatoma imaging, but in appropriate patients, higher resolution techniques may be preferable, so long as radiation doses are either comparable or lower than MDCT.

#### Contrast-enhanced CT (CE-CT)

CE-CT is not typically indicated for cholesteatoma imaging unless there is a suspicion of septic complications (such as an intracranial abscess or dural venous sinus thrombosis). It is particularly helpful in the emergency setting, but where clinically appropriate, contrast-enhanced MRI may be preferable (see below).

#### MRI

##### Standard structural sequences

Cholesteatomas are of high T2W signal and intermediate to low T1W signal. However, differentiation from other middle ear pathologies is limited on standard structural sequences. For instance, fluid, fibrosis, and granulation tissue may all have similar MRI characteristics in the context of CSOM, although the signal on T2WI and cisternographic sequences can be marginally lower in cholesteatomas when compared to fluid [15]. T1W imaging can aid discrimination of cholesteatoma from proteinaceous secretions and cholesterol granulomas, which demonstrate increased T1W signal, whilst high-resolution T2W sequences may provide an anatomical overview [16].

#### Intravenous gadolinium-based contrast

Cholesteatomas are non-enhancing, in contradistinction to enhancing middle ear substrates such as granulation tissue and fibrosis. However, the enhancement of fibrosis may be delayed due to poor vascularisation; therefore, delayed (45 min) post-gadolinium T1W imaging has historically been used to discriminate cholesteatoma from fibrosis (with non-enhancement or faint rim enhancement of the former and homogenous enhancement of the latter) [17, 18]. However, in contemporary practice, this technique is no longer applied since it is less convenient, specific, and reliable than DWI [17]. Instead, gadolinium-enhanced sequences are now typically only included in the setting of suspected infected cholesteatoma and associated complications.

#### Diffusion-weighted imaging (DWI)

Cholesteatomas demonstrate high signal intensity on DWI as a result of a combination of restricted diffusion and T2 shine-through effects [15]. Single-shot EPI (SS-EPI) DWI techniques are limited in their ability to image the middle ear due to a combination of poor resolution and artefacts (susceptibility, chemical shift and geometric distortion). As a result, other DWI techniques are typically employed (Table 1).

**Table 1:** DWI techniques used for cholesteatoma detection

Single-shot TSE DWI	
HASTE	⇒ Single-shot turbo spin echo sequence using a half-Fourier method to reduce the number of phase encoding steps. ⇒ Provides thinner section (2–3 mm) imaging. ⇒ It is resistant to distortion and susceptibility artefacts at the air-bone interfaces of the temporal bone [12]. ⇒ The single-shot technique and long echo train increase the risk of motion artefact and T2 blurring, but there is some mitigation by the half-Fourier acquisition [12, 13]. ⇒ Since only one b-value is acquired per acquisition, there is a requirement to acquire additional sequences in order to perform quantification of ADC values, which increases scan time and risk of motion artefacts [13].

Multi-shot TSE DWI	
Periodically rotated overlapping parallel lines with enhanced reconstruction (PROPELLER), BLADE, JET, MultiVane, depending on the manufacturer	⇒ *k*-space is acquired in a radial fashion with overlapping (and oversampling) at the centre. ⇒ The technique maintains a combination of high signal to noise ratio, reduced geometric distortions, lower motion sensitivity (due to shorter echo trains) and the ability to acquire 2 b-values in a single acquisition [12, 13].

Multi-shot EPI DWI	
Readout segmentation of long variable echo trains (RESOLVE)	⇒ The technique reduces echo spacing by dividing the k-space trajectory into multiple segments in the readout direction. ⇒ It provides high spatial resolution and thinner slices. ⇒ It is less sensitive to motion and geometric distortion than the single-shot technique and enables rapid acquisition of additional b-values [14].

#### Optimal DWI technique

**Fig. 4:**
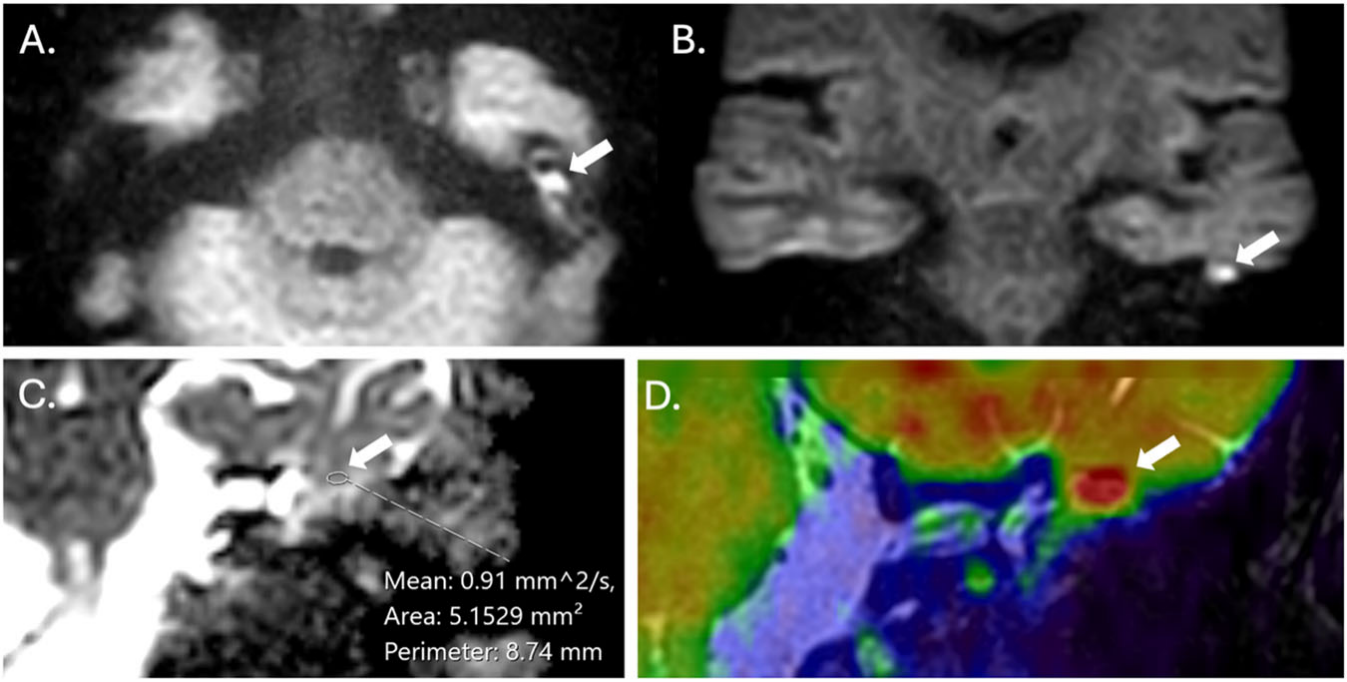
MRI of a left-sided middle ear cholesteatoma. Axial (**A**) and coronal (**B**) non-EPI DWI sequences through the temporal bones of a patient with a left posterior epitympanic cholesteatoma (arrows). The relationship with the middle cranial fossa is better depicted in the coronal sequence. Coronal ADC map (**C**) demonstrating restricted diffusion at the site of the cholesteatoma measured using an elliptical ROI (arrow). Coronal colourised coronal MRI fusion (between HASTE DWI MRI and T2 SPACE sequences) (**D**) highlighting the position of the cholesteatoma (arrow) relative to the lateral semicircular canal and tegmen tympani

Non-EPI DWI techniques significantly outperform SS-EPI in identifying residual or recurrent cholesteatomas, with higher sensitivities, specificities and negative predictive values [19, 20]. Whilst MS-EPI offers an alternative, there is evidence from single-institution studies that it results in greater distortion and artefacts with inferior diagnostic performance compared to non-EPI techniques [21–23]. DWI acquired in the coronal plane is often preferred as it enables more confident lesion localisation within the middle ear and mastoid (Fig. 4) [24].

#### Field strength

Non-EPI, MS-TSE, and MS-EPI DWI techniques can be performed at 1.5 T and 3 T field strengths. There has been some concern regarding increased artefacts due to magnetic field inhomogeneities at higher field strengths. However, modern hardware with improved B0 homogeneity can mitigate these drawbacks, and the improved signal-to-noise ratio at 3 T may be beneficial [25]. Single-institution studies have found scanning using multiple DWI techniques to be feasible at 3 T [23, 26].

#### Qualitative and quantitative assessment of ADC values

ADC values are calculated from DWI data to provide a quantitative metric of water diffusivity within each voxel. This can be of value in cholesteatoma imaging since cholesteatomas demonstrate lower ADC values than non-cholesteatomatous lesions, thereby improving specificity [27]. ADC maps can be assessed qualitatively with lower values demonstrated in cholesteatoma when compared to non-cholesteatomatous tissues; however, other temporal bone pathologies (e.g., abscess) may also result in reduced ADC signal and hence mimic cholesteatoma (Table 2) [28]. They can also be assessed quantitatively by drawing a region of interest (ROI) over the suspected lesion (Fig. 4). Several groups have sought to define thresholds for the diagnosis of cholesteatoma, but there is variation (range of 759–1370 × 10^−^^6^ mm^2^/s) due to the differences in DWI techniques and systems used [23, 27, 29, 30]. There are also challenges when defining regions of interest, particularly when lesions are small [23, 27]. Where there is uncertainty and lesions are equivocal, interval follow-up imaging can be helpful to establish lesion evolution over time.

**Table 2:** False positives (cholesteatoma mimics) on postoperative non-EPI DWI

**Substances that can return high signal on non-EPI DWI** [11, 14, 27]	⇒ Cerumen (seen in the EAC and mastoidectomy bowls, which can cause high false positive rates in CWDM). ⇒ Bone, cartilage, or soft tissue graft material. ⇒ Silastic. ⇒ Granulation tissue. ⇒ Cholesterol granuloma. ⇒ Scar tissue. ⇒ Encephaloceles. ⇒ Infection (exercise caution when middle ear/mastoid infection is suspected since increased DWI signal purulent fluid may mimic cholesteatoma).
**Top tip to avoid misdiagnosis**	⇒ Analyse DWI data alongside co-localised, unenhanced T1W sequences (proteinaceous mimics of cholesteatoma return a high signal in contrast to the low T1W signal of cholesteatoma) and high-resolution heavily T2W sequences (for an anatomical reference) [9].

#### High-resolution heavily T2-weighted sequences

**Fig. 5:**
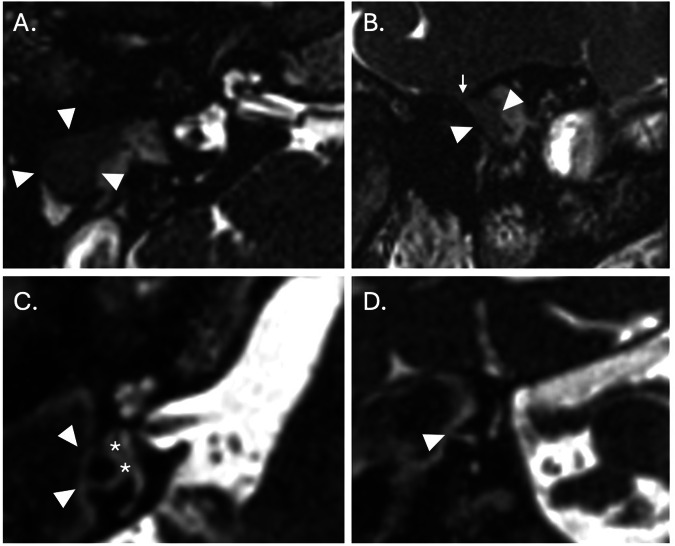
Cholesteatoma-mediated bone erosion on MRI. Axial (**A**) and sagittal (**B**) CISS MRI sections through the temporal bone of a patient who had previously undergone a CWUM for cholesteatoma. There is a defect in the tegmen mastoideum (small arrow) through which brain parenchyma (white arrowheads) herniates and demonstrates a lower signal than the surrounding middle ear fluid. Axial (**C**) and coronal (**D**) CISS MRI sections through the right temporal bone of a patient with an expanding cholesteatoma matrix causing erosion of the lateral semicircular canal (white arrowheads) and a reduction in perilymphatic signal locally (asterisks) in keeping with increased protein content

High-resolution 3D-gradient echo (such as constructive interference in steady state (CISS) and fast imaging employing steady-state acquisition-C) or 3D-fast spin echo T2W (such as Sampling perfection with application optimised contrast using different flip angle evolution (SPACE), Volume isotropic turbo spin echo acquisition (VISTA), and CUBE) sequences can be used to depict the inner ear fluid and adjacent cerebrospinal fluid spaces in the context of extensive cholesteatoma and help detect complications (such as labyrinthine fistulae, CSF leaks, and cephaloceles) (Fig. 5) [15].

#### Fusion of multimodality imaging

Fusion of non-EPI DWI data with high-resolution anatomical imaging results in a single dataset combining the advantages of both techniques (Fig. 4). This can increase the specificity and accuracy of localisation as well as predict suitability for endoscopic approaches [6]. Fusion of MRI sequences is technically simpler since patient positioning is unchanged, and fusion can be done using built-in software. Whilst CT fusion provides better delineation of bony structures, it is currently more time-consuming, requiring separate manual input to ensure accurate co-registration and incurs a radiation burden [6].

### Diagnosis

Typically, cholesteatoma is diagnosed on clinical and otoscopic evaluation, but CT can be employed where there is diagnostic uncertainty or when the EAC is occluded and otoscopy is not possible. It has an excellent negative predictive value if the middle ear and mastoid are well aerated without bone erosion [15]. However, false negatives can occur if the keratin has spontaneously evacuated, has been micro-suctioned, or where the typical erosion patterns are not seen. When the middle ear is opacified, it may be difficult to differentiate cholesteatoma from other middle ear substrates (such as granulation tissue or fluid). In such cases, identification of non-dependent middle ear opacification with a typical pattern of bone erosion (present in up to 50–97% of cases) or ossicular erosion (present in 82% of cases, with the incus most commonly affected) can increase diagnostic confidence [15, 31].

MRI is rarely required for routine preoperative diagnosis unless there remains diagnostic uncertainty following clinical examination and CT [32]. In particular, non-EPI DWI techniques are capable of identifying small (2–3 mm) lesions with a high degree of specificity [33]. MRI may also be helpful in differentiating high- from low-risk retraction pockets that cannot be adequately assessed otoscopically. In particular, higher non-EPI DWI signal in retraction pockets has been shown to correlate with the presence of cholesteatoma [34].

### Surgical planning and complications

CT can be considered in the routine preoperative workup of patients with cholesteatoma in order to determine lesion extent and to provide a surgical roadmap with a view to reducing the risk of iatrogenic injury to critical temporal bone structures [32]. This is particularly relevant when operating on a single hearing ear or when anatomy is aberrant due to congenital abnormalities or previous surgery [32]. It can also be used to assess the extent of erosion of the ossicles, labyrinth (usually the lateral semicircular canal), facial nerve canal, tegmen tympani, or sigmoid plate (Fig. 3) [32].

MRI is not normally routinely required for surgical planning but can be helpful in certain settings. For example, if erosion of the tegmen is suspected, 3D T2W sequences can be helpful in identifying intracranial extension or an associated cephalocele [33]. DWI MRI may localise and determine the extent of cholesteatoma, which may influence the type of surgical management in some centres; for example, demonstration of cholesteatoma extension beyond the posterior border of the lateral semicircular canal can contraindicate an endoscopic approach [6].

### Follow-up

#### Detection of residual cholesteatoma

**Fig. 6:**
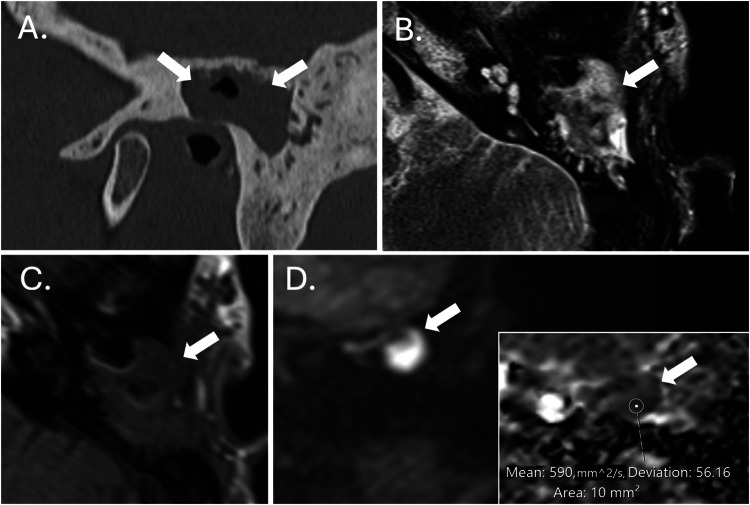
Follow-up imaging following a left CWUM. Imaging of the left temporal bone in a patient who had previously undergone a CWUM for cholesteatoma. The sagittal CT (**A**) shows nonspecific opacification of the mastoid cavity (arrows), which is expansile and extends above the EAC. On MRI, there is an area of increased signal on T2W (arrow on **B**), low to intermediate signal on T1W (arrow on **C**) and markedly increased signal on HASTE DWI (arrow on **D**) with low ADC values (590 × 10^−^^6^ mm^2^/s) (arrowed on inset image) compatible with residual cholesteatoma

**Fig. 7:**
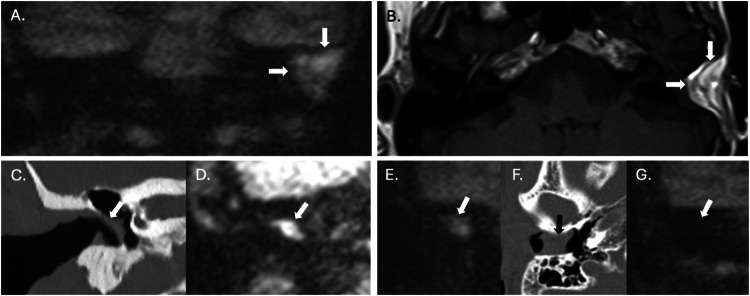
Mimics of cholesteatoma on non-EPI DWI. Coronal HASTE DWI (**A**) shows a large hyperintense area within the left mastoid (arrows). In the same patient, an axial T1W sequence through the temporal bones (**B**) reveals that the hyperintensity corresponds to fat-containing graft material used in a blind-sac closure (arrows). A coronal CT (**C**) demonstrates a cartilage graft used to reconstruct the right tympanic membrane (arrow), which is of high signal on coronal HASTE DWI (**D**), simulating a residual cholesteatoma (arrow). Coronal HASTE DWI through the right temporal bone of another patient (**E**) shows a hyperintense focus (arrow), which localises to the EAC (black arrow) on an axial CT (**F**) in keeping with cerumen. On a subsequent coronal HASTE DWI (**G**), the wax has been removed and the hyperintensity is no longer visible (arrow)

Non-EPI DWI techniques have become established as a cost-effective, non-invasive alternative to routine second-look surgery for residual cholesteatoma in patients who have previously undergone CWUM, with cholesteatoma demonstrating high signal relative to background tissues (Fig. 6) [35]. Systematic reviews and meta-analyses have demonstrated high pooled sensitivities, specificities and negative predictive values of at least 89.8%, 91.7%, and 80.5%, respectively, for the detection of residual cholesteatoma [19, 20, 36, 37]. DWI is also helpful in the context of other post-surgical situations where residual disease may be occult, such as previous mastoid obliteration or blind-sac surgery. It is worth noting that, in the postoperative setting, there are several structures that can return a high signal on non-EPI DWI studies and can therefore simulate residual cholesteatoma leading to false positives (Table 2 and Fig. 7). Additionally, where the risk of residual disease is high, such as extensive disease in paediatric patients, second-look surgery may still be planned (with or without a preoperative MRI) and can be combined with ossicular reconstruction [35]. CT is extremely limited in the detection of residual disease postoperatively since middle ear soft tissue changes (fluid, granulation tissue, and fibrosis) are frequent, nonspecific and cannot be reliably differentiated from cholesteatoma on CT (Fig. 6). Furthermore, it may be difficult to differentiate cholesteatoma-mediated erosion from surgical defects.

#### Duration and frequency of follow-up

Residual cholesteatomas have variable growth rates (2.7–4 mm per year), but may be more rapidly growing in paediatric patients and have been documented to occur late (5 years after surgery) [17, 38]. Therefore, postoperative surveillance imaging is indicated. The frequency of follow-up remains a matter of debate, but a suggested regimen is detailed in Table 3. Imaging intervals can also be varied in accordance with the perceived risk of recidivism. For example, when the initial MRI is indeterminate, repeat follow-up imaging in 1 year is often a useful strategy (particularly when the abnormality is not abutting critical structures).

**Table 3:** Postoperative MRI surveillance strategy

Timing of first postoperative MRI	⇒ > 12 months postoperatively for acquired cholesteatoma (scanning earlier risks a false negative scan in the setting of small (< 2–3 mm) residua). ⇒ > 18 months postoperatively for congenital cholesteatoma (unless the lesion is small and the matrix has not been violated, in which case routine follow-up imaging may not be needed) [30].
Frequency of follow-up	⇒ Assuming an initial negative study, perform an additional interval MRI 2–3 years postoperatively [38].
Duration of follow-up	⇒ Continue surveillance for at least 5 years [38].

#### Multidisciplinary working

Accurate interpretation of cholesteatoma imaging is greatly facilitated by collaboration with the multidisciplinary team. In particular, discussion with surgical colleagues is helpful to understand the nature of operative and reconstructive techniques used, along with the clinical context [24].

### Summary statement

**Fig. 8:**
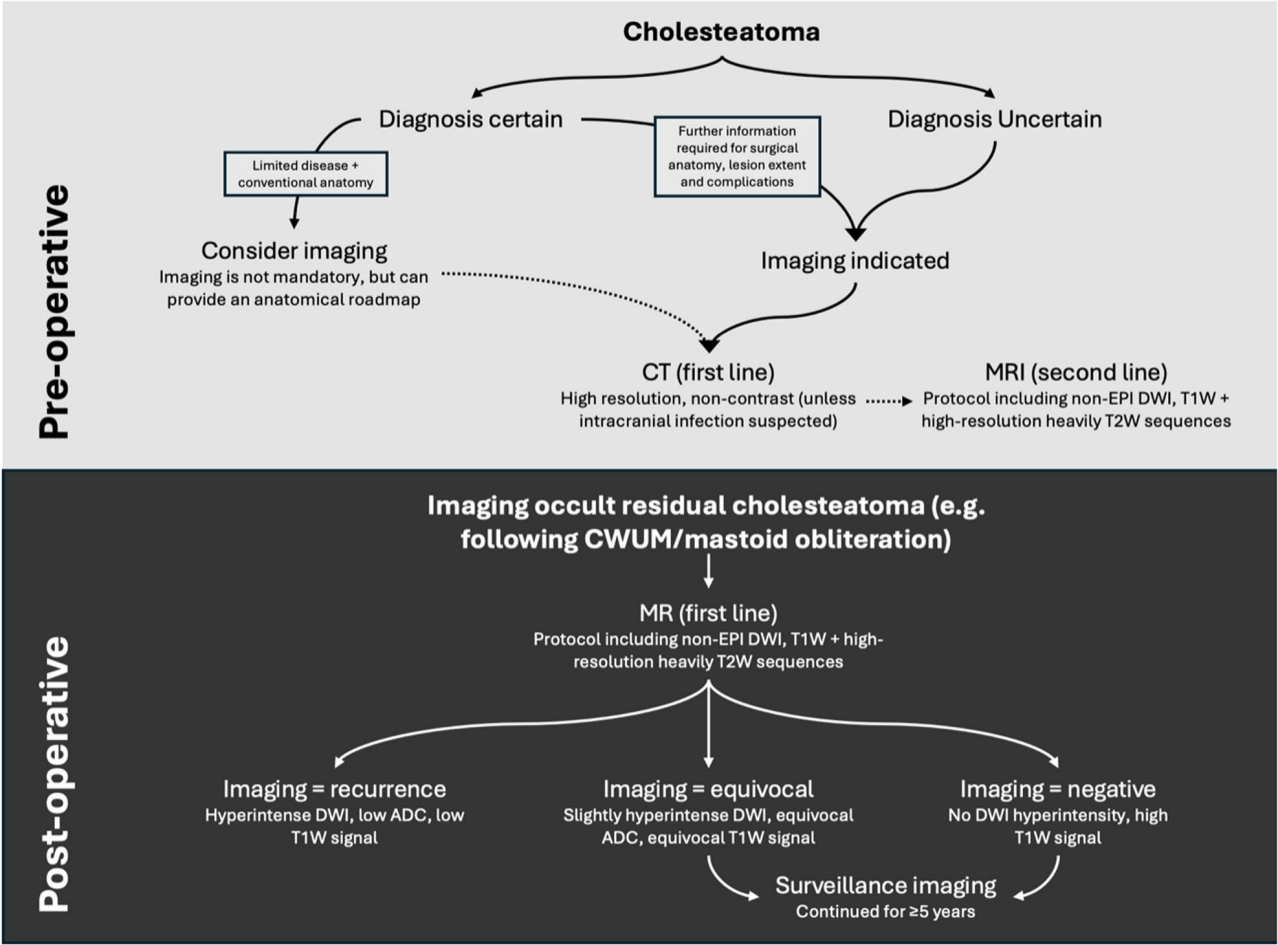
Flow chart indicating preoperative and postoperative imaging strategies for the evaluation of cholesteatoma

Imaging plays a significant role in the appropriate management of patients with middle ear cholesteatomas (Fig. 8). Whilst diagnosis is usually made clinically, CT can be helpful when otoscopy is not possible or for assessing lesion extent and erosive change. It also provides an anatomical roadmap prior to surgery, particularly where anatomy may be aberrant. MRI is not routinely indicated pre-operatively but can be used in specific situations. Following surgery, CT is typically limited due to its inability to distinguish residual disease from other soft tissue densities, but MRI is invaluable. Protocols that include non-EPI DWI can accurately detect residual cholesteatoma (unless very small < 2–3 mm). Supplementary structural MRI sequences improve specificity, provide anatomical landmarks, and detect complications. As a result, MRI surveillance represents an alternative to routine second-look surgery in patients who have undergone CWUM or obliterative or reconstructive techniques that would limit clinical detection of residual disease. Surveillance imaging is typically carried out at least 1 year after initial surgery and continued for at least 5 years.

### Patient summary

Cholesteatomas are non-cancerous growths that contain material related to the skin. They can grow deeply within the ear and damage local structures, so they are often removed surgically. Computed tomography may be used to help plan operations and to demonstrate the location and effects of cholesteatoma. New methods of magnetic resonance imaging have been shown to better depict cholesteatoma, and whilst further surgery was previously required for the detection of cholesteatoma regrowth, in many cases, magnetic resonance imaging can now be used instead. This document is directed towards helping radiologists make appropriate use of imaging techniques to aid patients with cholesteatoma.

## Abbreviations

ADC: Apparent diffusion coefficient
CISS: Constructive interference in steady state
CSF: Cerebrospinal fluid
CT: Computed tomography
DWI: Diffusion-weighted imaging
EAC: External auditory canal
EPI: Echo planar imaging
HASTE: Half-Fourier acquired single-shot turbo spin-echo
MRI: Magnetic resonance imaging
SPACE: Sampling perfection with application optimised contrast using different flip angle evolution
